# The presence of both serotonin 1A receptor (*HTR1A*) and dopamine transporter (*DAT1*) gene variants increase the risk of borderline personality disorder

**DOI:** 10.3389/fgene.2013.00313

**Published:** 2014-01-07

**Authors:** Peter R. Joyce, John Stephenson, Martin Kennedy, Roger T. Mulder, Patrick C. McHugh

**Affiliations:** ^1^Department of Psychological Medicine, University of OtagoChristchurch, New Zealand; ^2^Department of Health Sciences, School of Human and Health Sciences, University of HuddersfieldHuddersfield, UK; ^3^Department of Pathology, University of OtagoChristchurch, New Zealand; ^4^Division of Pharmacy and Pharmaceutical Sciences, School of Applied Sciences, University of HuddersfieldHuddersfield, UK

**Keywords:** personality disorder, depression, 5-HT, dopamine, genetics

## Abstract

Dysfunction in the dopaminergic and serotonergic neurotransmitter systems has been demonstrated to be important in the etiology of borderline personality disorder (BPD). We investigated the relationship of two BPD risk factors, the *HTR1A* promoter polymorphism -1019C > G (rs6295) and the dopamine transporter (*DAT1)* repeat allele, with BPD in a major depressive disorder cohort of 367 patients. Out-patients with major depressive disorder were recruited for two treatment trials and assessed for personality disorders, including BPD. DNA samples were collected and the rs6295 polymorphism was detected with a TaqMan^®^ assay. The *DAT1* repeat allele was genotyped using a modified PCR method. The impact of polymorphisms on BPD was statistically analyzed using uncontrolled logistic and multiple logistic regression models. BPD patients had higher frequencies of the *DAT1* 9,9 (OR = 2.67) and 9,10 (OR = 3.67) genotypes and also those homozygous *HTR1A* G allele (OR = 2.03). No significant interactions between *HTR1A* and *DAT1* genotypes, were observed; however, an increased risk of BPD was observed for those patients who were either 9,10; G,G (OR = 6.64) and 9,9; C,G (OR = 5.42). Furthermore, the odds of BPD in patients exhibiting high-risk variants of these two genes differed from those of patients in low-risk groups by up to a factor of 9. Our study provides evidence implicating the importance of the serotonergic and dopaminergic systems in BPD and that the interaction between genes from different neurotransmitters may play a role in the susceptibility to BPD.

## INTRODUCTION

Borderline personality disorder (BPD) is a psychiatric diagnosis in the Diagnostic and Statistical Manual of Mental Disorders, Fourth Edition (DSM-IV) manual that describes a prolonged disturbance of personality function characterized by depth and variability of moods. These disturbances can have a pervasive negative impact on many or all of the psychosocial facets of life. Attempted suicide and completed suicide are possible outcomes, especially without proper care and effective therapy (Black et al., 2004). Onset of symptoms typically occurs during adolescence or young adulthood. In the USA, BPD is estimated to affect 2% of the population [National Institute of Mental Health, (NIMH)]. Impulsivity and impulsive-aggression are core characteristics of patients with BPD. These are heritable traits of temperament associated with suicidal and aggressive behaviors in patients with BPD (Oquendo and Mann, 2000; Soloff et al., 2000a).

The etiology of BPD is still not clear. However, twin studies have found that if one identical twin met the criteria for BPD, the other also met the criteria in 35–69% of cases (Loranger et al., 1982; Torgersen, 2000; Zanarini et al., 2004; Distel et al., 2008b). Several studies have suggested that traits related to BPD are influenced by genes (Torgersen, 2000; Torgersen et al., 2000; Joyce et al., 2006, 2009; Distel et al., 2008a) including genes relating to serotonergic and dopaminergic neurotransmission. The dopaminergic system is involved in cognitive and emotional processing and the regulation of impulsive behaviors: dysfunction of these processes have been related to BPD symptoms (Friedel, 2004), which may stem from multiple factors, including a genetic component. Several studies have illustrated the role of dopaminergic genes in the etiology of BPD and associated traits, including the dopamine transporter (*DAT1*; Joyce et al., 2006, 2009; Giegling et al., 2008). Indeed, in a previous study, we demonstrated that the *DAT1* 9-repeat allele to be a significant genetic risk factor for BPD (Joyce et al., 2006). Furthermore, animal research suggests that repeated exposure to social stress has long-term effects on DAT1 density (Lucas et al., 2004) and that DAT1 knock-out mice demonstrate abnormalities of social interaction (Rodriguiz et al., 2004). These studies highlight the importance of the dopaminergic system, in particular DAT1, in the neurobiology of BPD.

Many studies have also reported serotonergic dysfunction in BPD including genetic, behavioral, and neuroimaging studies investigating neurobiological correlates in BPD individuals (Hollander et al., 1994; Soloff et al., 2000b, 2007; Leyton et al., 2001; Bennett et al., 2002; Paris et al., 2004; Retz et al., 2004; Gabbard, 2005; Ni et al., 2006a,b, 2009; Pascual et al., 2007; Zetzsche et al., 2008). Moreover, the effectiveness of SSRIs, such as fluoxetine, in the treatment of depression in patients with BPD lends support to the role that serotonin dysfunction plays in BPD (Goodman et al., 2004). Serotonergic autoreceptors, including the serotonin 1A receptor (*HTR1A*) have been linked to BPD (Hansenne et al., 2002; Ni et al., 2006a; Soloff et al., 2007; Zetzsche et al., 2008). Reduced HTR1A activity has been linked to BPD and suicidal behavior (Hansenne et al., 2002). Furthermore, a polymorphism in the *HTR1A* promoter region (rs6295) has been investigated in BPD through genetic and neuroimaging studies (Serretti et al., 2007; Zetzsche et al., 2008; Ni et al., 2009). Although two of these reports failed to find a clear link between the rs6295 polymorphism and BPD (Serretti et al., 2007; Ni et al., 2009), Zetzsche et al. (2008) found that in BPD patients with the G allele, there were significant structural changes in the limbic system when compared to individuals with a C,C genotype.

Although both serotonergic and dopaminergic pathways have been implicated in BPD, no previous genetic studies have examined the joint (interactive) effects of both the *DAT1* and *HTR1*A variants simultaneously on BPD. Due to the relationships of these neurotransmitter pathways with BPD etiology, we sought to determine if there was an interaction between the BPD risk factor polymorphisms, *DAT1* repeat allele and *HTR1A* 1019C > G (rs6295), to see if there was an increase in the risk of BPD. Genetic analyses were performed using a cohort of 367 major depressive disorder patients measured for personality disorders including BPD.

## MATERIALS AND METHODS

### SUBJECTS

Patients with a principal diagnosis of major depressive disorder were recruited for two depression treatment studies aged ≥18 years from a wide variety of sources, including mental health out-patient clinics, general practitioners, self-referral, and psychiatric emergency services. No advertising for patients was involved. Exclusion criteria were minimized, but included a history of schizophrenia or mania, a principal current diagnosis of severe alcohol or drug dependence, or severe current medical illness. In addition, patients needed to be free of psychotropic medication for a minimum of 2 weeks, or five drug half-lives, except for an occasional hypnotic for sleep. Patients also could not have received in the past 12 months an adequate treatment trial of one of the trial treatments to which they could be randomized. All patients were from New Zealand of European descent. In the first depression treatment trial, 194 patients were randomized to fluoxetine or nortriptyline (Joyce et al., 2002); and in the second trial 173 patients were randomized to interpersonal psychotherapy or cognitive behavior therapy (Luty et al., 2007), giving a total of 367 patients for the current study.

The two trials ran sequentially, in the same university-based out-patient Clinical Research Unit, and were the only depression treatment studies being undertaken in the Department at the time. Both treatment studies had been approved by the Canterbury (New Zealand) Ethics Committee. After being referred, patients were screened over the telephone by a research nurse who confirmed depressive symptoms and checked inclusion and exclusion criteria. Those who appeared suitable for inclusion were seen by a psychiatrist, senior psychiatric registrar, or clinical psychologist for an initial assessment. After giving consent, the patient then attended for a detailed clinical assessment. Assessment included the Structured Clinical Interview for DSM (SCID; Spitzer et al., 1992), which had been expanded to include assessment of all DSM-III-R and DSM-IV melancholic and atypical depression symptoms. Approximately 6 weeks after commencing treatment a trained psychiatrist or clinical psychologist completed the Structured Clinical Interview for Axis II Personality Disorders (SCID-II; Spitzer, 1987). Other clinician ratings were the Montgomery-Asberg Depression Rating Scale (MADRS; Montgomery and Asberg, 1979), the Hamilton Rating Scale for Depression (HRSD; Hamilton, 1960), and the Mental State Examination (MSE; Parker et al., 1994). An independent research nurse completed the MADRS, the HRSD, and the MSE. During the baseline assessment patients completed a series of self-report questionnaires, which included the Hopkins Symptom Checklist (SCL-90; Derogatis et al., 1973), the Structured Clinical Interview for Personality Disorders Questionnaire (SCID-PQ; First et al., 1997), the Parental Bonding Instrument (PBI; Parker, 1979) and the Temperament and Character Inventory (TCI; Svrakic et al., 1993). After this assessment patients were randomized to fluoxetine or nortriptyline in the first treatment trial (Joyce et al., 2002); and interpersonal psychotherapy or cognitive behavior therapy for the second treatment trial (Luty et al., 2007). However, in the current study, the two sample cohorts were considered to comprise a single sample, with no distinction made between the two cohorts, as the treatment variable in each cohort was not of direct interest and did not affect the relationship between allele status and the criterion variable of interest, BPD diagnosis. DNA was also collected for genetic studies.

## GENOTYPING

Genomic DNA was obtained from peripheral blood using sodium chloride extraction (Ciulla et al., 1988) and re-suspended in Tris–EDTA buffer (pH 8.0) and stored at -20°C until analysis. For all genotyping positive and negative controls were included. For *DAT1* repeat allele genotyping, data from a previous study was obtained (Joyce et al., 2006), but for validation purposes 25% of the sample were re-genotyped using a previously described polymerase chain reaction (PCR) method (Sullivan et al., 1997). The single-nucleotide polymorphism (SNP), located in the promoter region of the *HTR1A* gene rs6295 (1019C > G), was genotyped using a TaqMan^®^ probe (Applied Biosystems, Scoresby, Vic, Australia). One allelic probe was labeled with FAM dye, and the other with fluorescent VIC dye. PCRs were performed in a 384-well plate format within a total volume of 5 μl containing 2.73 μl of 2× ABgene Universal PCR Master Mix (Advanced Biotechnologies Ltd, Surrey, UK), 0.07 μl of 40× TaqMan^®^ probe (Applied Biosystems) and 20 ng of genomic DNA. Plates were placed in the LightCycler^®^ 480 real-time PCR System (Roche Diagnostics, Auckland, New Zealand), and heated at 95°C for 10 min, followed by 40 cycles of 95°C for 15 s, 60°C for 1 min and 72°C for 1 s. The fluorescence intensity in each well was measured and analyzed using the LightCycler^®^ (Roche Diagnostics). Genotypes were assigned using the end-point genotyping analysis software. The genotypes of 15% of the samples were verified by sequencing (Genetics Analysis Services, University of Otago). The web-based analysis program SHEsis^1^ (Shi and He, 2005) was used to check for deviations in Hardy–Weinberg equilibrium (HWE).

### STATISTICAL ANALYSIS

All data was entered into a relational database and subsequently transferred to the statistical package IBM SPSS (version 20.0) for statistical analysis. The sample was summarized descriptively to consider the incidence of BPD in various sub-groups of patients. Logistic regression analyses were conducted to investigate the association between BPD and the presence of the G allele of the rs6295 -1019C > G polymorphism; the presence of the* DAT1* 9-repeat allele; and the demographic factors of age and gender. Initially a series of uncontrolled analyses were conducted. Patients were classified according to their *HTR1A* genotype, with those homozygous for the C allele (subsequently referred to as the C,C variant) considered to be the reference category and those homozygous for the G allele (subsequently referred to as the G,G variant) and those heterozygous with the C and G alleles (subsequently referred to as the C,G variant) tested against the reference category. Patients were also classified according to the status of the 9-repeat allele of the *DAT1* gene, with the 10-repeat polymorphism on both alleles (subsequently referred to as the 10,10 variant) considered to be the reference category and the 9-repeat polymorphism on both alleles (subsequently referred to as the 9,9 variant), and the 9-repeat and 10-repeat polymorphisms on each allele (subsequently referred to as the 9,10 variant) being tested against the reference category. Four patients were found to exhibit the 11-repeat or 12-repeat polymorphism: these were deleted from the data set before further analysis.

Any factor appearing to be important from the uncontrolled analyses was considered for inclusion in a subsequent multiple logistic regression model. As the analysis was primarily concerned with associations between BPD and the *DAT1* and *HTR1A* genotypes in conjunction, these variables were forced into a multiple model. Statistical significance, odds ratio, and associated confidence intervals were estimated for each remaining parameter from this multiple model. Interactions between gene variants were also tested in this model.

## RESULTS

The two sample cohorts for this study were combined and consisted of a total of 367 patients (131 males, 236 females) with a mean age of 33.3 years (SD 10.9 years). There was no significant difference in the age distribution of males and females [mean male age 33.1 years (SD 11.0 years); mean female age 33.5 years (SD 10.9 years)]. The range of the data was 18–63 years in both males and females. Out of the full combined cohort, a valid diagnosis of whether or not the patient had BPD was recorded for 347 patients, with 50 (14.4%) having a positive diagnosis of BPD and 297 (85.6%) having no diagnosis of BPD. **Table 1** gives a summary of the frequencies of patients for whom the *HTR1A* genotypes were recorded, and for whom the *DAT1* genotype was recorded and validated from a previous study. The variants were in HWE (*HTR1A*, *p* = 0.52; *DAT1*; *p* = 0.77).

**Table 1:** The frequency of age, gender and DAT1 and HTR1A genotypes, with the prevalence of BPD.

Variable	Frequency (%)		
	BPD	No BPD	Total^1^
Age [mean (SD)]	29.5 (8.5)	34.4 (11.2)	33.3 (10.9)
**Gender**
Male	16 (4.4%)	115 (31.3%)	131 (35.7%)
Female	34 (9.3%)	202 (55.0%)	236 (64.3%)
All	50 (13.6%)	317 (86.4%)	367 (100%)
**Genotype**
*HTR1A*
C,C	8 (2.9%)	64 (23.1%)	72 (26.1%)
C,G	13 (4.7%)	131 (47.5%)	144 (52.2%)
G,G	12 (4.3%)	48 (17.4%)	60 (21.7%)
All *HTR1A*	33 (12.0%)	243 (88.0%)	276 (100.0%)
*DAT1*
10,10	15 (4.7%)	178 (55.5%)	193 (60.1%)
9,10	21 (6.5%)	88 (27.4%)	109 (34.0%)
9,9	5 (1.6%)	14 (4.4%)	19 (5.9%)
All *DAT1*	41 (12.8%)	280 (87.2%)	321 (100%)

*BPD, borderline personality disorder. ^1^Includes cases for which BPD status was not recorded. Percentages may not sum to 100% due to rounding.*

The relative frequencies of BPD in patients with certain allele combinations were compared. The incidence of BPD in patients with the 9,9, variant (five out of 19; 26.3%); or the 9,10 variant of the *DAT1* gene (21 out of 106; 19.8%) was 2.36 times greater than the incidence of BPD in patients with the 10,10 variant (15 out of 179; 8.4%). The incidence of BPD in patients with the G,G variant of the *HTR1A* gene (12 out of 60; 20.0%) was 1.75 times the incidence of BPD in patients with the C,C variant (eight out of 70; 11.4%) or the C,G variant of this gene (13 out of 136; 9.6%).*P*-values, odds ratios, and associated 95% confidence intervals (CIs) for all parameters considered in uncontrolled logistic regression models are summarized in **Table 2**.

**Table 2:** Results from an uncontrolled logistic regression models (all considered parameters) predicting BPD.

Variable	OR	95% CI for OR	*p*
Age	0.96	(0.93, 0.99)	0.004
Gender	1.13	(0.60, 2.14)	0.712
***DAT1* genotypes**
10,10 (reference)			
9,10	2.20	(1.13, 4.27)	0.020
9,9	2.47	(0.84, 7.27)	0.101
***HTR1A* genotypes**
C,C (reference)			
C,G	0.58	(0.28, 1.22)	0.153
G,G	2.20	(1.01, 4.79)	0.046
**Interactions**
(9,10 × C,G)	3.08	(0.58, 16.4)	0.188
(9,10 × G,G)	1.32	(0.27, 6.58)	0.735
(9,9 × C,G)	1.53	(0.10, 24.4)	0.762
(9,9 × G,G)	0.68	(0.04, 11.7)	0.792

*BPD, borderline personality disorder; OR, odds ratio; CI, confidence interval.*

The *Age* parameter exhibited a substantive or statistically significant association with the outcome and were carried forward for inclusion in a multiple logistic regression model, together with all indicator variables modelling the *DAT1* and *HTR1A* genotypes. Gender was not found to exhibit a substantive or statistically significant association with the outcome and was not considered in a multiple model. No interactions were found to be significantly associated with the outcome: following standard procedures, all interactions were removed and the model re-cast as a main effects model. The output from this model is summarized in **Table 3**.

**Table 3:** Results from a multiple logistic main effects regression model predicting BPD.

Variable	Adjusted OR	95% CI for OR	*p*
Age	0.95	(0.91, 0.99)	0.011
***DAT1* genotypes**
10,10 (reference)			
9,10	3.27	(1.42, 7.48)	0.005
9,9	2.67	(0.63, 11.3)	0.183
***HTR1A* genotypes**
C,C (reference)			
C,G	0.72	(0.27, 1.92)	0.511
G,G	2.03	(0.74, 5.58)	0.171

*BPD, borderline personality disorder; OR, odds ratio; CI, confidence interval.*

When controlling for other factors and covariates, age and *HTR1A, DAT1* variants were significantly associated with the incidence of BPD (**Table 2**). For *HTR1A*, a comparison of the reference category of the *HTR1A* gene with the other two categories (C,G and G,G) appears to show non-significance of the variant with BPD. The odds of the BPD in patients with G,G genotypes [2.02; 95% CI: (1.10, 4.79)] are significantly greater than the odds in patients who have the C,G genotype (0.58; 95% CI 0.28, 1.22): however, this comparison is not shown in **Table 2** as C,G was not selected as the reference category. Furthermore, the G,G genotype shows some degree of substantive significance with respect to the reference category of C,C (*p* = 0.171), while failing to reach statistical significance at the 5% level.

Higher incidences of BPD are found in younger patients, with the odds of BPD incidence reducing by about 5% for each year of age. Higher incidences of BPD are also found in patients with the 9,9 and 9,10 variants of the *DAT1* gene than in patients with the 10,10 variant. Similarly, higher incidences of BPD are found in patients with the G,G variant of the *HTR1A* gene than in patients with the C,G and C,C variants of this gene.

To explore the risk of BPD from both *HTR1A* and *DAT1* genotypes, the effect associated with specific combinations of these genotypes were considered. The effect of both the *DAT1* and *HTR1A* variants results in a compounding of odds ratios (**Table 4**). The highest odds of BPD occurred in patients homozygous with the G,G allele of the *HTR1A* gene and either the 9,10 (OR, 6.64) or 9,9 (OR, 5.42) of the *DAT1* gene. The lowest risk category, which was below the baseline risk of 1 or reference category (10,10; C,C), was 10,10; C,G. The risk ratio between the highest (9,10; G,G) and lowest (10,10; C;G) risk categories was 9.22. Conversely the effect of the presence of a particular variant of the *DAT1* gene may “cancel” the effect of another variant of the *HTR1A* gene: for example the odds of BPD occurrence in patients with the 9,10 variants of the *DAT1* gene, and the C,G variants of the *HTR1A* gene are within 18% of the odds of BPD occurrence in a patient with the reference categories of the 10,10 variant of the *DAT1* gene and the G,G variant of the *HTR1A* gene.

**Table 4:** Baseline risk of BPD (*DAT1* 10,10 and *HTR1A* C,C) = 1.00, with frequencies of gene combinations.

Risk categories	Frequency	Adjusted OR (95% CI)
10,10; C,C	44 (16.3%)	–
10,10; C,G	81 (30.0%)	0.72 (0.27, 1.92)
10,10; G,G	33 (12.2%)	2.03 (0.74, 5.58)
9,10; C,C	21 (7.8%)	3.27 (1.42, 7.48)
9,10; C,G	54 (20.0%)	2.35 (1.42, 3.88)
9,10; G,G	21 (7.8%)	6.62 (3.62, 12.12)
9,9; C,C	6 (2.2%)	2.67 (0.63, 11.27)
9,9; C,G	6 (2.2%)	1.92 (0.68, 5.44)
9,9; G,G	4 (1.5%)	5.40 (1.67, 17.4)

*BPD, borderline personality disorder; OR, odds ratio. Risk ratio between highest (9,10;G,G) and lowest (10,10;C,G) risk categories = 9.22.*

## DISCUSSION

In a previous study we identified that BPD was significantly associated with the *DAT1* 9-repeat allele variant (Joyce et al., 2006). In this study, the odds ratios associated with the *DAT1* 9-repeat allele (either 9,9 or 9,10) are consistent with our previous study, which was an analysis of a similar (but not identical) data set (Joyce et al., 2006). In the current study, however, the effect of the 9-repeat allele is considered as a controlling variable when assessing the effect of the *HTR1A* rs6295 allele in BPD. By contrast, the analysis of Joyce et al. (2006) focused on the *DAT1* allele as the key experimental variable and did not consider its effect in conjunction with the *HTR1A* allele.

The current study confirms that the *HTR1A* G allele increased the risk of BPD when controlling for the presence of the *DAT1* 9,9 variant. Two previous studies failed to find a positive link between the *HTR1A* G allele and BPD (Serretti et al., 2007; Ni et al., 2009), and although our study did not reach statistical significance, individuals with the G,G genotype showed an increase risk of BPD. The analysis also revealed a significant increase in BPD when the two risk alleles were present on both genes. The odds of BPD in patients in highest risk categories were those with the G,G variant of *HTR1A* and either 9,9 (OR, 5.42) or 9,10 (OR 6.64) genotypes of *DAT1*. Moreover, the odds of BPD in patients with the highest risk category (9,10; G,G) were about nine times greater than those in the lowest risk category (10,10; C,G). The sub-group including patients at highest risk had an incidence of BPD of 25.3% (18 out of 71): nearly double the overall incidence rate in the full cohort.

Neither the number of 9-repeats of the *DAT1* gene, nor the number of instances of the G allele in the *HTR1A* gene, seems to be additive effects. This suggests that a simpler model, in which the 9,10 and 9,9 variants of the *DAT1* gene are merged together, and the C,G and C,C variants of the *HTR1A* gene are merged together, may provide an adequate summary of the data. While both the 9,9 and 9,10 variants of the *DAT1* allele were found to be associated with BPD to a significantly greater extent than the 10,10 variant in an uncontrolled model, in a multiple model the incidence of BPD associated with the 9,9 variant was not significantly greater than the incidence of BPD associated with the 10,10 variant. This may be due to the relationships between gene variants.

One potential limitation of these findings is that the association has been found in clinical samples of depressed patients. In the community, the prevalence of BPD is about 1–2%, but in out-patient samples it is often 10–15% (Hyman, 2002). Mood disorders increase the risk of BPD. Any depression sample is thus an “enriched” sample, and it is possible that our findings in two combined depressed samples may not extend to the minority of BPD patients who do not suffer from depression. Another consideration is samples size and the possibility of false positives. However, with 367 patients, including 50 cases of BPD, the study was deemed adequately powered to detect any significant effects. Simulation studies by Peduzzi et al. (1996) suggest that with the number of positive cases, up to five variables may be considered in a multiple logistic regression model without compromising stability of parameter estimates. The sample size and number of positive BPD cases in our model is hence within acceptable limits.

Genetic factors contributing to BPD are thought to be only modest (Torgersen, 2000) and that the environment may have a significant impact on the development of BPD. However, twin studies of BPD have shown that 35–69% of the variance in BPD is caused by genetics (meaning that respectively 65% to 31% of the variance in BPD is caused by other factors, such as environment; Loranger et al., 1982; Torgersen, 2000; Zanarini et al., 2004; Distel et al., 2008b). This suggests that BPD has at least moderate genetic cause and is most likely an interaction of genes and environment leading to BPD. From our observations it is apparent that genetic factors contributing to BPD may be as a result of more than one risk variant, and consequently the involvement of more than one neurotransmitter system in traits underlying BPD. The complexity of BPD therefore cannot be delineated by single gene effects alone, which may explain, in part, why previous studies investigating the *HTR1A* G allele and BPD have been negative (Serretti et al., 2007; Ni et al., 2009). However, Zetzsche et al. (2008) revealed that BPD patients harboring the *HTR1A* G allele were significantly more likely to have a decrease in amygdala volume. The amygdala is part of the limbic system and controls negative emotion. Hyperactivation of the amygdala observed in BPD (Herpertz et al., 2001; Guitart-Masip et al., 2009), may reflect the heightened response to emotionally relevant environmental stressors in individuals with this disorder (Herpertz et al., 2001). Loss of inhibitory regulation in the amygdala results in disinhibited fear response, anger, and impulsive-aggressive behavior: core characteristics of BPD (Phillips and LeDoux, 1992). Promoter studies have shown that the minor G allele increases *HTR1A* activity (Czesak et al., 2012), and as an autoreceptor could conceivably reduce serotonergic function. Indeed, low levels of the serotonin metabolite, 5-hydroxyindoleacetic acid, have been detected in the cerebrospinal fluid of BPD patients (Brown et al., 1982). Reduced serotonergic function could conceivably inhibit the ability of this neurotransmitter system to modulate or control impulsive and aggressive behavior in BPD individuals. When combined with dysfunction in the dopaminergic system, potentially through DAT1, this could increase the susceptibility to BPD. Therefore, to fully understand the etiology of BPD, there is a clear need to interrogate multiple genetic factors and/or neurotransmitter systems in more depth.

## Conflict of Interest Statement

The authors declare that the research was conducted in the absence of any commercial or financial relationships that could be construed as a potential conflict of interest.

## Footnotes

1 http://analysis2.bio-x.cn/myAnalysis.php
